# DIAMOND (DIgital Alcohol Management ON Demand): a mixed methods feasibility RCT and embedded process evaluation of a digital health intervention to reduce hazardous and harmful alcohol use

**DOI:** 10.1186/s40814-017-0177-0

**Published:** 2017-08-21

**Authors:** Fiona L. Hamilton, Jo Hornby, Jessica Sheringham, Stuart Linke, Charlotte Ashton, Kevin Moore, Fiona Stevenson, Elizabeth Murray

**Affiliations:** 1 eHealth Unit, Department of Primary Care and Population Health, University College London, Upper 3rd Floor, Royal Free Campus, Rowland Hill Street, London, NW3 2PF UK; 2 Department of Applied Health Research, UCL, London, UK; 3 Camden and Islington NHS Foundation Trust, London, UK; 4 Camden and Islington Public Health, London, UK; 5 Institute for Liver and Digestive Health, UCL, London, UK

## Abstract

**Background:**

Alcohol is a major risk factor for preventable illness, with huge cost to healthcare economies. There is a role for alcohol-specific digital health interventions (DHI), but there have been few randomised controlled trials (RCT) comparing DHI with face-to-face treatment. Such trials are complex and face obstacles in recruitment and retention.

**Methods:**

Mixed-methods feasibility RCT of an alcohol DHI, testing recruitment, online data-collection and randomisation processes, with an embedded process evaluation. Recruitment ran from October 2014 for 9 months. Participants were adults drinking at hazardous and harmful levels, attending four community drug and alcohol services (CDAS) in London. Participants completed baseline demographic, alcohol-related and other psychological questionnaires online and were randomised to HeLP-Alcohol, a six-module DHI with weekly reminder prompts (phone, email or text message), which mirrors face-to-face treatment, or to face-to-face treatment at CDAS. Alcohol counsellors took part in qualitative interviews at the end of the study.

**Results:**

Alcohol counsellors screened 1253 patients. One thousand one hundred eighty-nine did not meet inclusion criteria so were excluded: 579 were dependent drinkers, 548 had health conditions that made them ineligible to take part and 62 were ineligible for other reasons including homelessness. Of the 64 patients who were eligible to take part, 54 declined to participate, with 36 stating a preference for face-to-face treatment, 13 gave no reason, and 5 gave other reasons including not wanting to use a computer. Ten consented but then 3 changed their minds, so we were able to randomise 7 participants to the study (11% of eligible).

Five alcohol counsellors agreed to be interviewed for the process evaluation and provided the following feedback: Although most of their colleagues were enthusiastic about the trial, some were not at equipoise in recruiting; potential participants also declared strong preference to intervention arm from the outset. These factors affected recruitment. Counsellors also lacked time to undertake the data inputting and follow-up of participants in addition to their everyday work.

**Conclusions:**

This feasibility study aimed to test recruitment, randomisation, retention and data collection methods but recruited only 7 participants so these aims were not fully achieved. This illustrates to all researchers of complex interventions the importance of conducting feasibility studies and is generalisable to areas other than alcohol research.

CDAS were seeing larger numbers of non-dependent drinkers with complex additional problems than alcohol commissioners expected. CDAS clients and some counsellors were not at equipoise for recruitment. Alternative settings for recruitment need to be explored in future trials.

**Trial registration:**

International Standard Randomized Controlled Trial Number: ISRCTN31789096, DOI 10.1186/ISRCTN31789096

## Background

Alcohol is a major risk factor for preventable illness. Worldwide, excessive use of alcohol is estimated to cause 4% of total mortality and between 4 and 5% of disability-adjusted life years [1–3]. Globally, around 4% of people drink more than recommended levels [4], but in England, up to 20% of the adult population drink heavily [5]. The resulting harm from alcohol has been estimated to cost England between £21 billion [6] and £47 billion [7] per year. The costs are attributed mainly to the large number of hazardous and harmful drinkers, as defined in Table 1, rather than the smaller number of dependent drinkers. Around 16.6% of the population in England drink at levels thought to be hazardous [5], and approximately 1.9% of the population drink at harmful levels. In contrast, about 1.4% of the population is estimated to be physically dependent on alcohol [8].

**Table 1:** Terms used to describe problem alcohol use

*Hazardous drinking* A pattern of alcohol consumption that increases someone’s risk of harm (WHO) [66]
*Increasing risk drinking* An alternative term for hazardous drinking, mainly used in the UK, defined as ‘Regularly consuming between 21 and 50 units a week for men and between 14 and 35 units a week for women’, [67] with a unit being equivalent to 8 g of pure alcohol. [68]
*Harmful drinking* A pattern of alcohol consumption that is causing mental or physical damage (WHO) [66]
*Dependent drinking* A cluster of behavioural, cognitive and physiological factors that typically include a strong desire to drink alcohol and difficulties in controlling its use. [67]
*Higher risk drinking* An alternative term for harmful and dependent drinking, defined as ‘Regularly consuming over 50 alcohol units per week for men or over 35 units per week for women’. [67]

Reducing excess consumption of alcohol is an NHS priority and government policy aims to address alcohol-related harm through alcohol screening and brief intervention (SBI), also known as identification and brief advice (IBA) [9, 10]. IBA is a ‘spend to save’ public health policy which, along with controlling pricing, availability and marketing of alcohol [11], aims to shift population consumption downward, leading to overall lower levels of morbidity and NHS spending [12], although some have questioned the effectiveness of this policy [13].

In England, people who continue to drink heavily despite IBA can also be referred to Community Drug and Alcohol Services (CDAS). CDAS provide open access treatment conducted by specialist alcohol counsellors, ideally with a comprehensive substance misuse assessment, alcohol-specific information, advice and support, and brief interventions based on motivational interviewing [14]. Treatment typically takes place weekly for 6 to 8 weeks based at CDAS, although there may be shared care with general practitioners.

Only a small proportion of people who could benefit from treatment actually access it, for a range of reasons, including low levels of identification in primary care, stigma and ongoing underfunding of services [15–17]. Those who do attend often drop out of treatment due to confusing care pathways and the need for high levels of motivation to engage with the care offered [18]. There is therefore an urgent need for effective and cost-effective alternatives to face-to-face treatments. Digital health interventions (DHI) may bridge the gap in treatment provision for people with non-dependent levels of drinking who do not require supervised detoxification.

DHI are programmes that provide information and support for behaviour change or to manage physical or mental health problems via a digital platform such as a website [19]. The NHS Forward View [20] recommends increasing investment in prevention (including help for people with alcohol problems), utilising innovative ways to provide healthcare, including technology, and to ‘exploit the information revolution’ to do this. The marginal costs per new user of DHI are relatively low; hence, DHI could improve access for services where there are shortages of staff. DHI may overcome worries about stigma as they can be accessed privately, are convenient to use, do not interfere with work commitments and can be revisited if a person needs additional help in the future, for example, in preparation for a high-risk event (such as a birthday or other celebration), or if the user feels at risk of relapse [21]. This is particularly important for conditions like alcohol misuse where relapse is a frequent problem [22].

DHI are easily accessible, as around 84% of people in Great Britain have access to a computer or smart phone [23], and 78% of adults (39.3 million) accessed the internet every day, or almost every day, in 2015 [24]. DHI solutions may introduce inequalities for the minority of people who do not use the internet, and these people are likely to be older, less well-educated and from the most-deprived communities [25–27]. However, if some groups take up DHI preferentially, this may free up resources to undertake targeted face-to-face interventions for hard-to-reach groups such as these. In addition, DHI are subject to attrition (defined as non-use or sub-optimal use of the intervention [28]).

A recent systematic review suggests that the use of DHI may be an effective and cost-effective way of treating hazardous and harmful alcohol use [29]. However, most RCTs included in the systematic review compared DHI with websites that assessed control participants’ alcohol consumption levels and then provided generic feedback or just gave information about harmful effects of excessive use of alcohol. It is important to conduct trials comparing the web intervention against the ‘gold standard’ of face-to-face treatment by a specialist alcohol counsellor.

Alcohol trials are complex, for several reasons. People with alcohol problems may be difficult to recruit and retain [30–34] due to denial of having a problem, embarrassment, having other commitments during the day, or drinking so heavily so they forget appointments or cannot complete outcome measure questionnaires online or in paper form [35]. To try to overcome the problem with recruitment, we planned to recruit from a pool of people attending CDAS for the first time for help with their drinking. These people evidently are motivated to seek treatment so have overcome some of the barriers to potential recruitment. However, going to an alcohol service takes a great deal of motivation, and people may experience a range of reactions to being offered online treatment when they expect to see a counsellor for face-to-face treatment. Alcohol counsellors themselves may have strong feelings about referring clients to receive online treatment.

We wanted to elicit the views of alcohol counsellors and trial participants to identify the facilitators and barriers to taking part in the trial. There have been few qualitative research studies published exploring the experiences of either clients or counsellors taking part in Internet trials of alcohol interventions. Qualitative studies have so far mainly focused on users’ opinions about the content and functionality of Internet interventions [36, 37].

For these reasons, we undertook a feasibility study and an embedded process evaluation to learn more about the challenges likely to be encountered in a fully powered phase 3 trial. The trial was planned and co-designed with the input from local alcohol commissioners, taking into account local treatment models for non-dependent drinkers, to ensure it addressed questions of policy relevance, maximised acceptability of trial procedures and ensured smooth fit with existing workflows [38], and could provide data which would inform practice.

Specific objectives were

1. To estimate recruitment rates to a phase 3 RCT
2. To examine retention to the trial
3. To test online randomisation and data collection instruments
4. To collect data to inform the sample size calculation for the main RCT
5. To understand the reasons for any difficulties in recruitment and retention to the trial, and with data collection or use of the DHI

Although recruitment and retention rates would give an impression of the acceptability to both clients and counsellors of offering online treatment, the mixed methods approach using qualitative interviews would give us much richer information about how people feel about being offered online treatment when they attend a community alcohol service for help, whether an online intervention is acceptable to alcohol counsellors, and would also give valuable feedback for developing the intervention and the trial processes for the definitive trial.

## Methods

We conducted a randomised controlled feasibility trial with an embedded process evaluation. The methodology for the feasibility study is described briefly below, with more detail in the published protocol [39].

### Patient public involvement (PPI)

In order to ensure that the setting was appropriate for recruitment, we sought the views of patient representatives. Alcohol counsellors from each participating CDAS were asked to approach clients who had previously used their service to see if they would be interested in helping the researchers to develop the study design and recruitment materials. Three patients were interested in being patient representatives for the study and had ongoing involvement in iterations of the trial design, and one patient representative attended trial management group meetings They also took part in ‘think aloud’ testing of the online alcohol intervention. In think aloud testing, users describe their immediate reactions to using the DHI while the researcher observes how they use it [40]. Their feedback was used to adapt and improve the intervention. A separate group of patient representatives user tested the trial recruitment portal, the participant information leaflet (PIL) and consent form, all of which were adapted in the light of their feedback.

In addition to PPI input, we also had significant and meaningful input from other stakeholders, including commissioners, who we approached to determine whether CDAS would be an appropriate place to recruit. Their commissioning specifications included services for hazardous and harmful drinkers so they were certain that recruitment would be possible from these sites. The alcohol commissioners, along with alcohol service providers, helped to refine the trial procedures including inclusion / exclusion criteria, recruitment and follow-up.

### Setting

The setting was in four Community Drug and Alcohol Services in inner London.

### Participants

People aged ≥ 18 years drinking at hazardous and harmful levels (AUDIT score ≥ 8 [41, 42]) attending a participating CDAS for their first appointment, able to use a computer, and not having any of the exclusion criteria listed below. Hazardous drinkers are those drinking more than recommended limits for drinking (14 units a week or 2–3 units daily in the UK) [43] and who are at risk of, but not yet experiencing, alcohol-related harms. Harmful drinkers are people drinking more than recommended limits and experiencing alcohol-related harms but without symptoms of physical or psychological dependence.

### Exclusion criteria

Dependent drinkers (Leeds Dependence Questionnaire [44] score ≥ 20) having a serious mental health conditions such as schizophrenia or bipolar disorder, being at risk of self-harm or suicide, or currently undergoing treatment for substance use disorder; having a serious physical health problem (e.g. liver disease, cardiovascular disease, cancer); having legal issues likely to lead to imprisonment; being homeless; having child protection issues; being a victim or perpetrator of domestic violence; being pregnant; not being able to speak English; not being able to use a computer.

### Recruitment

Recruitment for the feasibility study took place in two stages. First, local CDAS were recruited and counsellors were trained in recruitment. Secondly, participating CDAS alcohol counsellors recruited patients to the study. At the end of the study participants and counsellors were invited to take part in the process evaluation.

1. CDAS recruitment: the alcohol commissioners involved in designing the study recommended five local CDAS to approach. All were interested in taking part and we visited each to present the proposed study at team meetings. The alcohol counsellors and administrative staff gave feedback on the proposed methods at these visits and helped to develop the design of the study to fit with their working patterns. For example, they suggested the following changes: to exclude people who were potentially at risk of suicide or for whom there might be safeguarding or child protection concerns; CDAS to be responsible for weekly contacts for those participants randomised to the DHI, as a duty of care, rather than the trial manager as originally proposed: all participants randomised to the DHI should be advised to return to the alcohol service following the web intervention unless they decided they did not wish to do this. Each CDAS identified a principal investigator (PI) and a second counsellor to help the PI lead the research. From June 2015, CDAS staff were trained in Good Clinical Practice (including trial paperwork, confidentiality, data management/clinical governance) and supported in recruiting and consenting participants, by a trial manager. After the Web-developers finalised the trial portal and website, recruitment started in October 2014 and ran for 9 months.
2. Participant recruitment: new clients were assessed by CDAS staff as per their usual practice. If a client met the eligibility criteria for the study the alcohol counsellor discussed the trial with them, and gave them a participant information leaflet (PIL) if they were interested. The potential participant signed the consent form and had a 24-h cooling off period before being contacted by the counsellor again. If still happy to participate, their details were uploaded by the counsellor to the trial website portal and an email was sent to the participant with a link to complete baseline questionnaires before being individually randomised by computer to the DHI or to treatment as usual (TAU), face-to-face treatment with an alcohol counsellor. The participant was then sent an email with instructions for accessing the intervention website or for making an appointment with the CDAS.
3. Process evaluation: we invited all alcohol counsellors involved in the study and all participants who completed follow-up measures. We had considered asking clients who had been eligible but declined to take part in the feasibility study, but this was not in the original ethics application and by the time we had ethical approval to approach them they had left the service and their contact details deleted.

The CDAS PIs and other alcohol counsellors were invited by email to take part in the qualitative interviews after recruitment to the feasibility RCT ended. The feasibility RCT participants were invited to take part after completing final follow up measures online: the website displayed a screen thanking them for taking part in the feasibility study with a request to click a link to indicate they were interested in taking part in further research.

The counsellors and trial participants who responded to the invitations were then emailed a PIL and consent form for the qualitative study. They were then contacted to arrange a convenient time and date for the interview.

### Intervention

This was an online alcohol treatment programme for hazardous and harmful drinkers called Healthy Living for People who use Alcohol (HeLP-Alcohol). The programme is not suitable for dependent drinkers due to the risks of sudden cessation such as seizures [45]. HeLP-Alcohol was developed from an automated online alcohol treatment programme called Down Your Drink, which mirrored, as far as possible, treatments known to be effective face-to-face at community alcohol services [21, 46, 47]. It had three phases: the first phase was based on motivational interviewing techniques, aiming to encourage the user to reach a considered decision about changing drinking behaviours; the second phase on cognitive behavioural therapy (CBT) and behavioural self-control techniques to help users cut down; the third phase focused on relapse prevention [21]. HeLP-Alcohol also had an online drink diary, and users could set treatment goals and record their thoughts and feelings in response to the various modules. They were also able to set up their own text message reminders, e.g. to help them avoid drinking too much in social situations. There were films of dramatised case studies for each module to maintain interest and engagement. Information about alternative local and national sources of support was also provided.

Participants randomised to this arm received a weekly email or 10-min phone call (depending on user preference) from a named facilitator to promote engagement with the intervention [48, 49]. The contacts were made by administrative staff who did not provide any alcohol-related therapy. They were briefed just to remind users to login to the website, and to provide help if they were having problems using the website.

### Comparator

The comparator was face-to-face treatment as usual (TAU) in four community drug and alcohol services (CDAS) in north London. Each CDAS provided treatment in a tailored way: individual face-to-face sessions with a counsellor, and/or group sessions, with the option of attending complementary therapies at some services, e.g. yoga, gardening. Some CDAS counsellors saw patients at their GP surgery. As this was a feasibility study rather than an efficacy study we did not try to standardise TAU across the services.

### Outcome measures

As per the CONSORT extension to randomised pilot and feasibility trials, [50] the primary outcomes were feasibility outcomes and secondary outcomes included patient centred data collection.

#### Primary outcome measures

The following feasibility outcomes were collected:

1. Recruitment as a percentage of eligible patients.
2. Retention measured by completeness of online data collection for each arm at baseline and at 1 and 3 months as a percentage of patients randomised, also giving an indication of the acceptability of each arm.

#### Secondary outcome measures

The following outcomes for both study arms were collected via online questionnaires at baseline and follow-up are shown in Table 2. They are all self-report measures.

**Table 2:** Secondary outcome measure questionnaires

Item	Description	Collected at baseline	Collected at 1 month	Collected at 3 months
Demographic characteristics	Age, sex, ethnic group, highest educational attainment and area deprivation (measured by Index of Multiple Deprivation [69])	**✓**	**✗**	**✗**
LDQ	Leeds Dependence Questionnaire, a 10-item questionnaire [44]	**✓**	**✗**	**✗**
Unit consumption of alcohol per week	TOT-AL, an online beverage-specific measure [22] which requires participants to enter the type and quantity of alcohol drinks consumed on each day of the past week	**✓**	**✓**	**✓**
AUDIT	Alcohol Use Disorders Identification Test [6], a 10-item questionnaire developed by the World Health Organization to identify problem drinking	**✓**	**✗**	**✓**
CORE-10	Clinical Outcomes in Clinical Evaluation questionnaire, [23], a 10-item questionnaire to measure current psychological global distress score developed and validated as a non-proprietary measure of psychological distress	**✓**	**✗**	**✓**
SCQ-8	Situational Confidence Questionnaire, [25], an 8-item questionnaire to measure confidence in avoiding alcohol in a range of situations	**✓**	**✗**	**✓**
CSQ-8	Client Satisfaction Questionnaire, an 8-item questionnaire developed to measure satisfaction with care provided by mental health services, [26, 27] and also used for assessing satisfaction with alcohol and other substance misuse programmes [28]	**✗**	**✗**	**✓**
Attendance	Whether participant attended CDAS or used HeLP-Alcohol at 1 month	**✗**	**✓**	**✗**
Adherence to the intervention (for those randomised to this arm),	Measured through automated recording of numbers of log-ins and numbers of pages visited at each log-in	**✗**	**✗**	**✓**
Other sources of support accessed during treatment	Using a drop down menu of options: group therapy, horticulture, acupuncture, art therapy, other therapies (participant to state in free text)	**✗**	**✓**	**✓**

##### Data collection for the feasibility study

1. Recruitment

The alcohol counsellors completed weekly recruitment logs, which they sent to the trial manager. They recorded the number of new clients accessing the service, how many fulfilled the eligibility criteria, how many were asked to take part and how many agreed to take part. When clients declined to take part and offered a reason, alcohol counsellors recorded this reasons and included it in the recruitment log. The numbers of participants who ultimately logged on, completed baseline questionnaires and then were randomised were recorded automatically, along with the data on baseline measures.

2. Retention

Participants were emailed requests to complete follow up measures online after 3 months, with a £10 shopping voucher being offered to complete the outcome measures. Using a monetary incentive has the strongest evidence to support its effectiveness in increasing completion rates in trials [51–53]. Retention data were collected automatically from the participants who completed online follow up measures. The usage data for participants randomised to HeLP-Alcohol were automatically captured by the website. The website developers were compliant with Good Clinical Practice and transferred all data to the researchers in an anonymised format.

##### Data collection for the process evaluation

Interviews took up to 1 h, with open questions based on a topic guide. One pilot interview was held prior to interviewing participants to finalise the questions and ensure smooth running of the study. Interviewees took part in the study in their own time and were offered a token of gratitude (a shopping voucher for £25). Each conversation was audio-recorded.

All identifying details were removed when the interviews were transcribed, and each participant was assigned a unique number. The recordings were stored digitally on university computers until professionally transcribed and were then securely deleted.

### Sample size

As this was a feasibility study there was no formal sample size calculation. There is not consensus on the number of participants required for a feasibility study, but Teare et al. suggest around 35 in each arm is sufficient [54]. As drop out rates of up to 84% are possible in online alcohol trials [47, 55], we aimed to recruit 100 for each arm in order to reach at least 35 in each arm at follow-up. We proposed to interview 10 alcohol counsellors and up to 20 trial participants for the embedded process evaluation.

### Analysis of secondary outcome quantitative data

As this was a feasibility study, we aimed to collect data for the primary outcome (previous week’s alcohol intake in units) for each arm so that the effect size (change in alcohol intake) could be calculated with measures of variance for the subsequent sample size calculation.

### Analysis of qualitative data

The qualitative data were collected to complement and aid in the interpretation of the quantitative data generated by the feasibility study [56]. Transcripts were coded by hand, and thematic analysis was used to examine the transcripts. The use of thematic analysis enables new insights to inform the subject guide for subsequent interviews and earlier transcripts to be revisited throughout the process of coding and theme allocation [57].

## Results

### Recruitment and retention

**Fig. 1:**
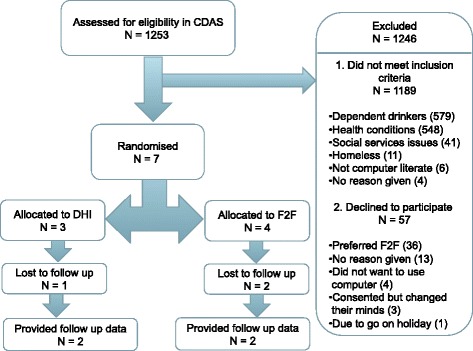
CONSORT flowchart for recruitment and retention to the DIAMOND feasibility RCT. Abbreviation: F2F face-to-face

Alcohol counsellors screened 1253 patients, of whom 1189 did not fulfil the inclusion criteria so were excluded from the study: 579 were dependent drinkers and 548 had other health problems (co-morbid drug use or severe physical or mental health problems); 41 had child protection or domestic violence issues; 11 were homeless; 6 were not computer literate and for 4 the counsellors did not specify the reason. Of the 64 eligible patients, 54 declined to participate: 36 preferred face-to-face treatment; 13 offered no reason; 4 did not want to use a computer; and 1 was due to go on holiday. 10 people initially consented but then 3 changed their minds, so overall, 7 participants were randomised, 11% of eligible: 3 to HeLP-Alcohol and 4 to face-to-face TAU group, and we were able to collect follow-up data on 4 of these, with 3 lost to follow-up; 1 from the HeLP-Alcohol group and 2 from face-to-face group. For a detailed breakdown of these results, see the CONSORT flowchart in Fig. 1.

Ten alcohol counsellors were invited to participate in the qualitative study and five agreed to take part, two male and three female. Further demographic details were collected but not reported here to maintain confidentiality. There were too few interviews for robust themes to emerge, but the insights from the interviews are given below.

Three participants expressed interest in taking part. However, there was a delay of several months in obtaining additional ethical approval for the qualitative study (the initial approval for the feasibility study did not include interviews with NHS patients) and unfortunately none of these people responded to further email invitations to take part once ethical approval was received.

#### Reasons for participating

Alcohol counsellors were interested in taking part in the feasibility study to explore the role of DHI for alcohol treatment.

“Well I felt really good about being in the study, it was quite exciting and interesting, and you know an additional dimension and dynamic to the role.” Participant 151,126

“I felt it was very positive, felt quite interesting, and I think as a team we all felt very enthusiastic about how it was going to turn out.” Participant 160,301

#### Reasons for not recruiting at CDAS and patient level

The counsellors’ initial positive views of the trial were tempered by their frustration that the majority of service users were not eligible to take part, as they were dependent drinkers or had other complex problems or safeguarding issues such as domestic violence, or suicidality. They felt that even for the small number of eligible clients there were further barriers to participation. For example, many declined to take part as they were unfamiliar with computers or did not have access.

“I think it is about personality. It’s about telling, selling, getting the right person who’s got access to a computer, for one, and fulfils the criteria. Because quite often it was straightaway: not fill the criteria. There was a few, not very many, in our team meetings who fulfilled it to start with. And once they fulfilled it there’d be another hurdle, computer access or illiterate.” Participant 160,301

The counsellors also noticed that potential participants declared strong preference to either face-to-face or online intervention, and were reluctant to take part in the study because they might not get their treatment of choice. They thought it would be better to give participants the choice.

“It’s a big step, to come to an alcohol service, and a lot of the time then, people have already had a look online. So once they’ve come here, they probably do, a lot of the time, expect or want to see somebody who’s here in front of them.” Participant 151,211

“So it was just a bit frustrating that we’d got them on board and had pitched it and they were eligible, and they didn’t get onto the online. So the first one was really disappointed, he was really looking forward to it, felt like it would really suit him, suit his workflow, suit his schedule. You do all that work and then they don’t get to do the online programme anyway. So that may be a reason why people stopped selling it….So it would be better if there was the option there.” Participant 160,304

In addition, some counsellors lacked confidence with computers themselves and struggled to explain the intervention, or were suspicious of the long-term goals of the trial.

“Some people who, I think, weren’t really confident with online stuff themselves, or with the computer themselves. People who didn’t understand the trial and didn’t feel confident enough to explain it to other people. And people who knew the client group, were probably quite skeptical of something like this.” Participant 160,304

“I could see a lot of resistance within the teams where people would commonly say ‘oh well they want to replace us with computers.’….and then in retrospect you thought, hmmm, is that why your client sort of declined?” Participant 1,551,126

Some members of their teams were not at equipoise in recruiting, they held the opinion that some clients would not manage online, and if randomised to online would be ‘lost’ to the service, so did not offer them the option of being in the trial.

“It’s not the easiest thing to then sort of send somebody away, because we as a service would need to be very sure that that person isn’t presenting any kind of risk, or there’s not something we’ve overlooked… so erring on the side of caution, we might then decide to keep the larger proportion of people here.” Participant 151,211

Other members of the team were unenthusiastic about the extra work the trial presented, with data inputting and follow up of participants in addition to their everyday work. Some of the trial methods were confusing or onerous or introduced delays. It was difficult for alcohol counsellors to know if someone had been randomised to the website or if had just dropped out of attending the service, and this resulted in a lot of chasing up on the part of the counsellors.

“There’s so much to do in the assessment, my concern at the start was when do we do this? When do we ask this question? When do we get them to fill out….? So logistically, how is this going to work, really?” Participant 160,304

Counsellors also felt the trial would have benefitted from local senior management support for the trial, taking into account the extra work involved. They felt lack of support impacted on the motivation of alcohol counsellors to recruit to the trial.

“The hierarchy did not seem to support [the trial]…I think it was very much, you know, this has been agreed by commissioners, service managers were told what to do, not really signed up for it, not really research focussed.” Participant 151,126

#### Reasons for not retaining at patient level

Long-term drinkers accessing CDAS often have very chaotic lives and tend to disengage with the service after a few sessions, either because they start drinking again, or because their lives start to improve, or they are busy, and so the counsellors were not surprised that they dropped out of the trial.

“Hugely common. I mean, many of our patients drop out, that’s the most common picture for us, is you see them once, it’s all very hopeful, they’re really interested, and then you’ll never see them again.” Participant 160,304

#### Suggestions for changes to the design and intervention

The counsellors made a number of suggestions for improving the trial, but the consistent theme was to take into account client preference in the design. They also thought that it would be better to have dedicated administrators and research associates responsible for recruitment and follow up, preferably alcohol counsellors or other healthcare professionals. It was difficult for counsellors to undertake these tasks in addition to their usual workload. They also felt that the weekly phone call prompts to participants randomised to HeLP-Alcohol did not work due to call screening (this could be because the CDAS number was not recognised, or because the number was withheld, or they did not wish to speak to CDAS staff.

Counsellors’ thoughts on how and where to recruit participants for a future study included workplaces and gyms, social groups used by retired people, university settings, online screening websites such as Don’t Bottle It Up [58], and hospital settings where patients get health screening. There were mixed views about recruiting from general practice as the counsellors felt that it was difficult for their client group to get appointments, and the GPs themselves are too busy to actively recruit participants, but a trial could be advertised in practice waiting rooms or research associates could recruit from waiting rooms.

### Online randomisation and data collection instruments

Online randomisation and data collection appeared feasible, although with such small numbers these outcomes were not robustly tested. All the participants completing baseline measures, although only four completed the 1- and 3-month follow-up measures, with three being lost to follow up. As so few participants completed follow-up measures, it was not possible to use the data for a sample size calculation for a phase 3 study.

The HeLP-Alcohol usage data for those randomised to this arm was automatically captured, and showed that one participant did not log in at all, one logged in three times on 1 day, and one participant accessed 159 pages over 4 days, so they may have only looked through rapidly without taking in the information.

## Discussion

The low numbers of participants recruited in the feasibility study suggested that community alcohol services were not suitable sites to recruit to a phase 3 RCT, because the majority of clients attending these services were dependent drinkers or had additional problems that excluded them from participating. Although it is well known that people attending for alcohol treatment have high rates of co-morbidity, e.g. up to 85% have a co-existent mental health problem, up to 60% are also misusing drugs) [59, 60], we did not expect there would be so many people who would be ineligible to take part. We had discussed the proposed study with alcohol commissioners and CDAS staff and they were confident that we could recruit sufficient numbers. In retrospect, we could have asked to examine CDAS records to see the profile of their patients and determine the proportion of dependent drinkers and hazardous and harmful drinkers with other conditions.

Another challenge was that from the small pool of potentially recruitable patients, the majority declined to participate, as they preferred to have face-to-face treatment. This meant that the number recruited was too small to draw any conclusion about the intervention’s effect on alcohol consumption so the data could not be used for the sample size calculation for a future RCT.

The qualitative interviews with the alcohol counsellors suggested that an alcohol DHI may be suitable for certain clients, those who are too embarrassed to attend services, those with work or have childcare commitments, and those with access to and familiarity with computers or smart phones. These findings are similar to those seen in a qualitative study by Khadjesari et al. [61]. Counsellors also thought that clients tended to have a strong preference for either face-to-face or online treatment from the beginning, and this affected both recruitment and retention in the trial. This finding supports previous work analysing recruitment to alcohol and other mental health trials [62, 55], which found that a major reason for non-participation was fear of being allocated to a placebo treatment. However, the HeLP-Alcohol DHI was an active treatment that closely mirrors face-to-face treatment, so we did not expect such a strong aversion to taking part in our study. On the other hand, as accessing treatment is challenging [18], once someone with problem alcohol use has finally navigated the system and made a face-to-face appointment, it is understandable that they may prefer to continue to be seen in person. Some team members themselves were not at equipoise with recruiting otherwise eligible clients and may have presented the study in such a way that the clients did not want to take part, or may not have asked them in the first place. This is not unique to either the clinical area or the professional group, as problems with recruitment due to equipoise have been also been found in trials of other treatments such as for heart conditions or urological procedures, with recruitment by doctors, nurses and researchers [63, 64]. Despite the hard work and enthusiasm of the PIs in trying to maximise recruitment, other counsellors may not have been as motivated to recruit to the trial due to the extra work the trial processes generated when they were already under huge time and work pressures. So having a specific member of staff to recruit participants, following initial assessment by the alcohol counsellors, could improve recruitment. However, the chaotic and ambivalent interactions CDAS clients typically have with services, and the effects of heavy drinking, may be the main reasons for the difficulties with recruitment and with retention and engagement with HeLP-Alcohol, as Ratke et al. have also found in their study with University students [35].

### Strengths and weaknesses

The feasibility trial struggled to recruit, but nevertheless succeeded in answering conclusively the research question: it is not feasible to undertake a large trial recruiting from CDAS units, and a different recruitment strategy is needed for a definitive trial. This finding was surprising as local alcohol commissioners had commissioned a service for harmful and hazardous drinkers as well as dependent drinkers, so it was reasonable to expect a higher proportion of clients would be eligible to take part in our study than was actually the case. We feel that for commissioners this new understanding of the people accessing the services they are commissioning is an important finding.

The high drop out may have been in part because the full set of follow-up questionnaires may have taken up to 30 min to complete, which may have been off-putting. Although questionnaire length as opposed to relevance was not found to affect retention in an alcohol study by McCambridge et al. [65], the small sample in our study meant that we were unable to see any fundamental problems with the data collection instruments.

The interviews with alcohol counsellors provided insights into the difficulties that alcohol trials often encounter with recruitment and retention due to client-, recruiter- and system factors. The main weakness of the qualitative study was that we were only able to interview five alcohol counsellors and none of the original trial participants. Although the insights from the five interviews will be useful in refining any future study, as all the counsellors interviewed had taken on the role of PI in the study, and were quite senior, the views of other counsellors may have differed from theirs.

## Conclusion

This feasibility study struggled to recruit participants and so was not able to fully test recruitment, randomisation, retention and data collection methods. This is an important lesson for other researchers who may face similar problems in recruiting to trials of complex interventions and is generalisable beyond the context of alcohol studies. Our findings illustrate the importance of conducting feasibility trials ahead of fully powered RCTs.

We found that CDAS are seeing large numbers of hazardous and harmful drinkers with additional health and social problems. Although this made them ineligible to take part in the study, it is a useful and finding for the commissioners of alcohol services. The reasons people from our target group did not present in sufficient numbers for our study are not clear from our data, but likely to include referral bias (referral of the sickest) and acceptance bias (patients not accepting help until they were very unwell).

We explored the barriers to recruitment through qualitative methods and found that both patients and counsellors were not at equipoise for recruitment, and some counsellors had underlying concerns about computers replacing face-to-face treatment in the long term. Trial processes were onerous for busy alcohol counsellors. The effects of streamlining trial methods and using alternative settings such as hospital clinics, or a more suitable point in the referral pathway from which to recruit such as general practice, need to be explored in future trials.

## Abbreviations

AUDIT: Alcohol use disorders identification test
CDAS: Community Drug and Alcohol Services
CONSORT: Consolidated Standards of Reporting Trials
CORE: Clinical Outcomes in Clinical Evaluation
CSQ: Client Satisfaction Questionnaire
DHI: Digital health intervention
DIAMOND: DIgital Alcohol Management ON Demand
HeLP-Alcohol: Healthy Living for People who use Alcohol
IBA: Identification and brief advice
LDQ: Leeds Dependence Questionnaire
NHS: National Health Service
NRES: National Research Ethics Committee
PI: Principal investigator
PIL: Participant information leaflet
PPI: Patient public involvement
RCT: Randomised controlled trial
SBI: Screening and brief intervention
SCQ: Situational Confidence Questionnaire
TAU: Treatment as usual
TOT-AL: TOTal ALcohol
WHO: World Health Organization
